# AsHC 360 Exposure Influence on Epileptiform Discharges in Hippocampus of Infantile Male Rats In Vitro

**DOI:** 10.3390/ijms242316806

**Published:** 2023-11-27

**Authors:** Lei Dong, Ling Zhao, Lei Tian, Wenjun Zhao, Chan Xiong, Yu Zheng

**Affiliations:** 1 School of Life Sciences, Tiangong University, Tianjin 300387, China; dong_lei@tiangong.edu.cn (L.D.); 2031101126@tiangong.edu.cn (L.Z.); tianlei@tiangong.edu.cn (L.T.); 2031101130@tiangong.edu.cn (W.Z.); 2 Institute of Chemistry, NAWI Graz, University of Graz, Graz 8010, Austria

**Keywords:** arsenic-containing hydrocarbons, hippocampus, epileptiform discharges, electrophysiological, in vitro

## Abstract

Arsenic-containing hydrocarbons (AsHCs) are typical arsenolipids found in various marine organisms. They can penetrate the blood–brain barrier, specifically affecting synaptic plasticity and the learning and memory ability of hippocampal neurons. Temporal lobe epilepsy often occurs in the hippocampus. Thus, the possible influence of AsHCs exposure to temporal lobe epilepsy garnered attention. The present study investigated the effects of epileptiform discharges (EDs) signals introduced by low-magnesium ACSF in the hippocampus of infantile male rats in vitro, using electrophysiological techniques with multi-electrode arrays under AsHC 360 exposure. In our study of the effects of AsHC 360 on EDs signals, we found that inter-ictal discharges (IIDs) were not significantly impacted. When AsHC 360 was removed, any minor effects observed were reversed. However, when we examined the impact of AsHC 360 on ictal discharges (IDs), distinct patterns emerged based on the concentration levels. For low-concentration groups (5, 20, 60 μg As L^−1^), both the frequency and duration effects on IDs returned to normal post-elimination of AsHC 360. However, this recovery was not evident for concentrations of 100 μg As L^−1^ or higher. IDs were only observed in EDs signals during exposures to AsHC 360 concentrations up to 60 μg As L^−1^. In these conditions, ID frequencies significantly enhanced with the increased of AsHC 360 concentration. At high concentrations of AsHC 360 (≥100 μg As L^−1^), the transition from IIDs or pre-ictal discharges (PIDs) to IDs was notably inhibited. Additional study on co-exposure of AsHC 360 (100 μg As L^−1^) and agonist (10 nM (S)-(-)-Bay-K-8644) indicated that the regulation of EDs signals under AsHC 360 exposure could be due to directly interference with the α-amino-3-hydroxy-5-methyl-4-isoxazole-propionic acid receptor (AMPAR) expression which influences the binding of excitatory glutamate neurotransmitter to AMPAR. The results suggest that EDs activities in the hippocampus of infantile Sprague Dawley rats are concentration-dependent on AsHC 360 exposure. Thus, it provides a basis for the seafood intake with AsHCs for epileptic patients and those with potential seizures.

## 1. Introduction

Arsenic is a metalloid element widely distributed in the form of inorganic arsenic (iAs) and organic arsenic [1]. Long-term exposure to iAs can damage the central nervous system and elevate the risk of neurodegenerative diseases [2]. Organic arsenic is rich in species, primarily distributed in microorganisms, plants, and marine organisms [3]. For instance, fish oil contains rich arsenolipids, [4,5] mainly comprising arsenic-containing hydrocarbons (AsHCs), arsenic-containing fatty acids (AsFAs) and arsenic-containing phospholipids (AsPLs) [6,7].

Recent studies have observed that AsHCs also exhibit high cytotoxicity and potential neurotoxicity. The concentration range causing cytotoxic effects is similar to that shown by iAs (the IC_50_ value of arsenite (As(OH)₃) is 5.2 μM for UROtsa cells and 17 μM for HepG2 cells), [8,9] and they have been found to have toxic effects on various cells such as cultured human urothelial cells (UROtsa, the IC_50_ value of AsHC 360 is 7.4 μM for UROtsa cells), hepatoma cells (HepG2, the IC_50_ value of AsHC 360 is 8 μM for HepG2 cells), pre-differentiated human neurons (the EC_30_ values on cell viability of arsenite and AsHC 360 are 6.3 μM and 2.2 μM), and fully differentiated human neurons (the EC_30_ values of arsenite and AsHC 360 are 6 μM and 7.3 μM) [9,10,11,12]. The known AsHCs include AsHC 332, AsHC 360, and AsHC 444 [13,14]. Among them, AsHC 360 possesses the highest cellular bioavailability and interferes with cellular membrane integrity [9,11]. A similar study was conducted to evaluate the effects of AsHCs exposure on long-term potentiation (LTP) of synapses in the hippocampal CA1 region. It was found that it enhanced the LTP under 3.75–15 μg As L^−1^ AsHC 360 exposure, while inhibited LTP was observed under 45–150 μg As L^−1^ AsHC 360 exposure [15]. Moreover, it has been demonstrated that AsHCs can be ingested by lactating women through seafood and transferred to infants through breast milk. The median total arsenic found in breast milk is around 1 μg As kg^−1^, of which up to 61% is lipid-soluble arsenic. Interestingly, the transportation efficiency of AsHCs is 3%, while only 0.03% of arsenobetaine is transported to the mother’s milk through dietary ingestion [16,17]. Since the developing nervous system is highly vulnerable to toxic substances, leading to impaired brain function [18,19,20], it is crucial to evaluate the abnormal activity of excitatory and inhibitory neurons in the hippocampus of the brain that may aroused by AsHC exposure.

Arsenic exposure remains a global public health concern, affecting millions of people through contaminated water and food sources. While direct experimental or clinical evidence linking arsenic or arsine exposure to seizures and epilepsy may initially be lacking, studying the effects of arsine exposure on epileptiform activity holds significant clinical and scientific implications. AsHC is known to have complex and multifaceted neurotoxic effects, a comprehensive investigation of neurotoxicity related to AsHC exposure entails the exploration of various neurochemical and physiological processes in the brain. The intricate interactions among these processes can be exceptionally challenging. However, extensive research has already been conducted on the neurotoxic principles underlying epileptic seizures or epileptiform activities. By comparing the results of this experiment with studies on drugs used to treat epilepsy or exposure sources that induce epileptic activity, we can gain a clearer understanding. This comparison allows us to refine the neurotoxic mechanisms of action, both in direct and indirect pathways, and parameters that significantly affect brain function. Consequently, it can help reveal new mechanisms or interactions that may be associated with arsenic exposure, thereby playing a crucial role in clinical risk assessment, diagnosis, and the management of individuals exposed to arsenic.

In our study, we evaluated six different concentrations of AsHC 360, namely 0, 5, 20, 60, 100, and 200 μg As L^−1^. The low concentration of 5 and 20 μg As L^−1^ for AsHC 360 exposure were selected based on a previous study where enhanced LTP effects were observed under exposure to 3.75–15 μg As L^−1^ of AsHC 360 [15]. It is important to note that we do not possess the accumulation factor of AsHCs in the human body (specifically infants). However, prior studies have indicated that arsenite and diphenylarsinic acid (DPAA) can pass through the blood–brain barrier and accumulate in brain tissue. Notably, a DPAA concentration of 600 μg As kg ^−1^ was discovered in the central nervous system, whereas only 21.2–46.5 μg As kg ^−1^ was found in the urogenital system, believed to be the main excretion pathway for arsenic. Consequently, the potential accumulation of AsHC 360 in the nervous system must also be taken into account [21,22,23,24]. Therefore, the chosen accumulation factors of 5–100 to be tested in our experiment are reasonable. The highest exposure level (200 μg As L^−1^) of AsHC 360 was determined based on previous studies that demonstrated the ability of AsHCs to cross the blood–brain barrier. Müller et al. observed that AsHC 332, AsHC 360, and iAs^III^ at a concentration of 10 μM were non-toxic during 6 h of blood–brain barrier penetration in vitro. However, AsHC 360 was highly susceptible to barrier integrity and local morphological abnormalities were observed in cells treated with AsHCs at a concentration of 2.5 μM (equivalent to 188 μg As L^−1^), including cell swelling and intercellular space [9]. These effects are concerning, as the cell membrane integrity and the ion channel status on the membrane are closely associated with epileptiform activity maintenance. Therefore, the highest exposure concentration used in this study was 200 μg As L^−1^ to ensure that all experiments were conducted without signal weakening or disappearance due to toxicity.

Temporal lobe epilepsy (TLE) is refractory focal epilepsy, [25] characterized by synchronous or abnormal discharge of brain neurons, which often originates in the CA3 region of the hippocampus [26,27]. Through in vitro and in vivo animal epilepsy models, it was determined that temporal lobe epilepsy begins in the CA3 area of the hippocampal horn, and the generated electrophysiological signals are gradually transmitted to the DG and CA1 areas to form local abnormal discharges [26,27]. At the same time, they are transmitted to the amygdala on the same side, even to the entire limbic system and the contralateral brain area, ultimately forming a group of epileptic discharges. According to the epidemiological survey, the annual prevalence rate of epilepsy in the general population is 5~7‰, and the performance has different effects on all ages, with obvious bimodal distribution characteristics. One peak is between 5 and 9 years old, and the other is around 80 years old. The incidence rate of children is 10~15 times that of adults. Childhood is also the initial onset period of most epilepsy patients. Therefore, focusing on the prevention and treatment of epilepsy in infants and children has always been a key research direction in neuroscience and other related fields. Several studies have indicated that arsenite can inhibit the regeneration of hippocampal neurons in vitro [28,29], suggesting that arsenic exposure may affect epilepsy activity through intervention in the CA3 region. In addition, exposure to sodium arsenite was found to suppress the level of glutamate receptor 1 and damage newborn neurons in the primary culture of mouse cortical neurons (at concentrations greater than 1 mM) [30]. Subunits GluA1 through GluA4 can assemble in various tetrameric combinations to constitute an AMPAR, which is crucial for neuronal discharge. Studies have also shown that exposure to iAs during pregnancy can alter the abundance of neurotransmitters of glutamatergic synapses and lead to changes in cysteine/glutamate transport, which is associated with the down-regulation of N-methyl-D-aspartic acid receptor (NMDAR) NR2A and NR2B subunits in the hippocampus in vivo [30,31]. Therefore, the effect of AsHCs exposure on the expression of excitatory neurotransmitters in the hippocampus should be investigated to better understand its potential impact on epileptic-like discharge activity.

In this study, we employed an established epilepsy model using Mg^2+^-free artificial cerebrospinal fluid (ACSF) on hippocampal slices from 8- to 12-day old Sprague Dawley rats. The study will record changes in epileptiform discharges (EDs) in the hippocampal CA3 region using the MEA2100-60 system. Additionally, the study will expose the epileptic model to multi-concentration AsHC 360 to assess its potential mechanism of affecting EDs signals. Furthermore, pharmacological experiments will be conducted to decipher the possible effect of glutamate receptor expression on EDs signals.

## 2. Results

### 2.1. Mg^2+^-Free ACSF Induces Normal EDs

**Figure 1:**
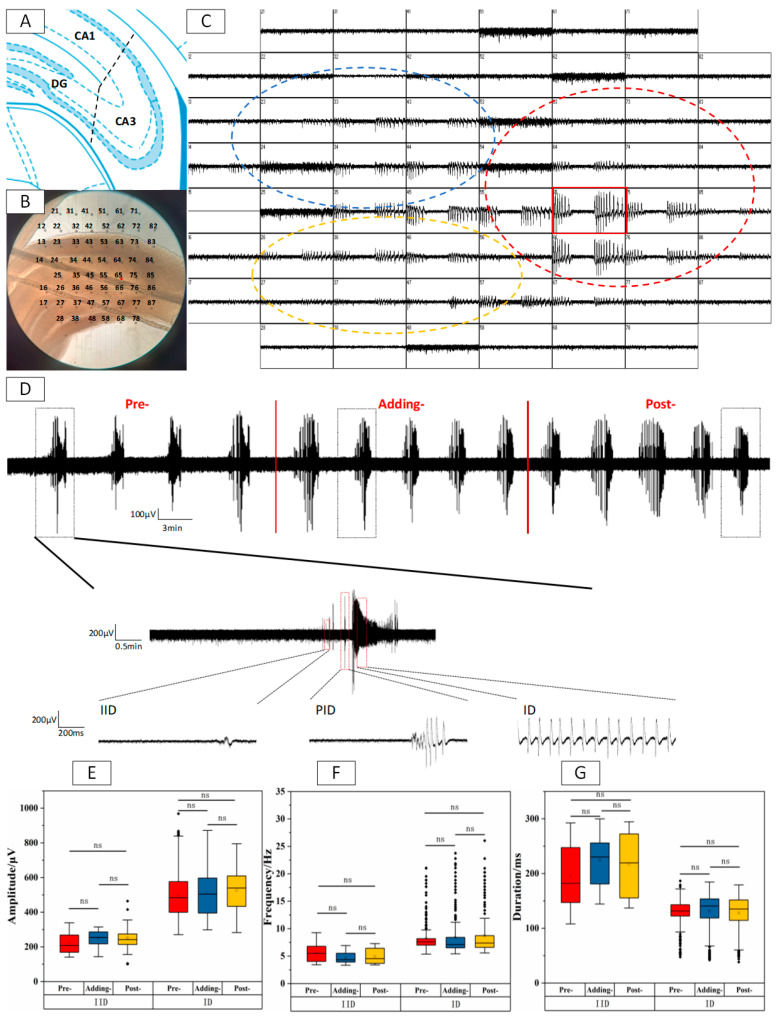
EDs within the CA3 region of hippocampal slices induced by Mg^2+^-free ACSF. (**A**) Distribution of CA1, CA3, and DG divisions of the hippocampal slices. (**B**) Real microscopic images (10 × 10) of each subregion of the hippocampal slice and the 64 points glass electrode. (**C**) EDs signals were recorded by 64 MEA points (Red ellipse: CA3 signals; blue ellipse: CA1 signals; yellow ellipse: DG signals). (**D**) Long-term records and representative state of 65# pole, such as single cluster EDs of Pre- and Post-stages. (**E**) Boxplots of IIDs and IDs amplitude within Pre-, Adding- and Post-stages. (**F**) Boxplots of IIDs and IDs frequency within Pre-, Adding- and Post-stages. (**G**) Boxplots of IIDs and IDs duration within Pre-, Adding- and Post-stages. “ns” represents no significant difference.

The schematic map in Figure 1A demonstrates the CA1, CA3, and Dentate Gyrus (DG) division across the hippocampal brain slice. The relative positions of each partition and 64 recording points of a typical hippocampal isolated brain slice were observed under the microscope (Figure 1B). Multi-point EDs were recorded in Figure 1C. In a previous study, EDs signals recorded in the CA3 region were the earliest and the strongest, suggesting that CA3 is the initiation region of EDs [32]. Therefore, the CA3 area was selected for data recording and analysis for subsequent experiments. A continuous EDs signal intercepted from CA3 (electrode point 65) is depicted in Figure 1D. A single-cluster ED can be divided into three phases, such as IIDs (inter-ictal discharges), PIDs (pre-ictal discharges), and IDs (ictal discharges), based on the continuous discharge time (Figure 1D). At this point, discharge events with a continuous discharge time of ≤300 ms are defined as IIDs, which are short and intense, mainly consisting of single peak spike discharge; the discharge events with a continuous discharge time of 300~2000 ms are defined as PIDs, they mainly consist of single peak and rhythmic oscillatory discharges; discharge events with a continuous discharge time of ≥2000 ms are defined as IDs, with discharge duration exceeding 5 s, mainly characterized by continuous rhythmic spike wave oscillation discharge. The discharge process also involves the conversion from IIDs/PIDs to IDs. Statistical analysis was conducted on amplitude, frequency, and duration on IIDs and IDs of EDs events in Pre-, Adding-, and Post-stages. The boxplots are shown in Figure 1E,G, and the original data have been listed in Table S1.

No significant difference (* *p* > 0.05, *n* = 5) in the amplitude, frequency, and duration of EDs between the Pre-, Adding- and Post-stages were observed in both IIDs and IDs phases. The results indicate that Mg^2+^-free ACSF could induce stable and long-term EDs within the hippocampal slices. Considering that AsHC 360 was preserved and tested by blending with Glycerin/H_2_O, a pre control experiment of Glycerin/H_2_O was conducted, and the results showed that Glycerin/H_2_O had no significant effect on the experiment. Please refer to Supplementary Document S3 for specific results.

### 2.2. The Effect of AsHC 360 Exposure on EDs

**Figure 2:**
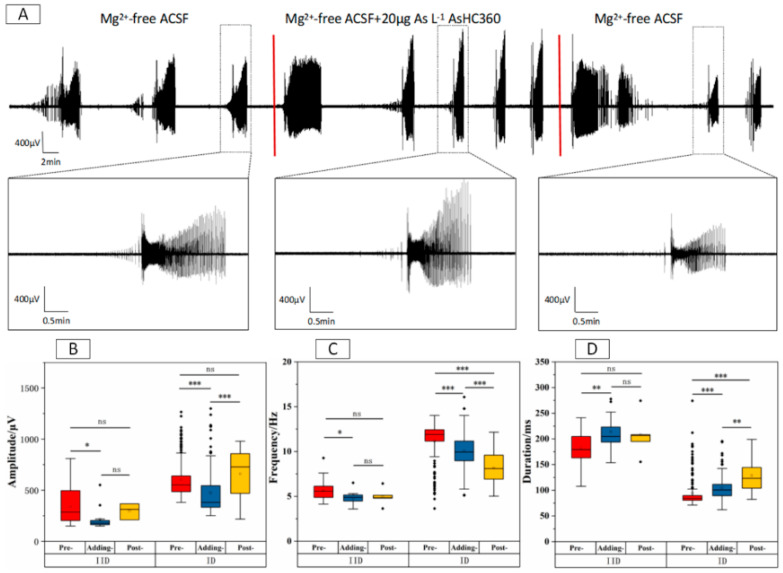
EDs within the CA3 region of hippocampal slices under 20 μg As L^−1^ AsHC 360 exposure. (**A**) Long-term records and representative ED clusters. The boxplots of IIDs and IDs (**B**) amplitude, (**C**) frequency, and (**D**) duration within Pre-, Adding, and Post-stages. “ns” represents no significant difference, “*” indicates *p* < 0.05, “**” indicates *p* < 0.01, and “***” indicates *p* < 0.001.

**Figure 3:**
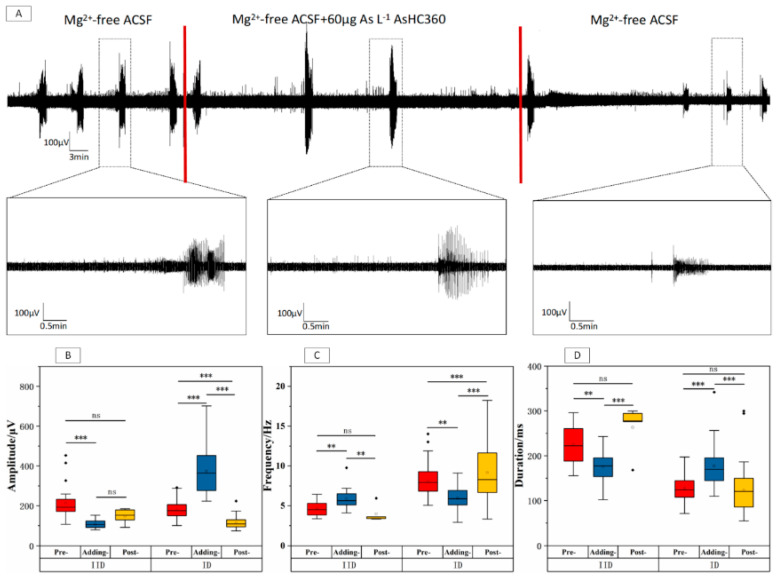
EDs within the CA3 region of hippocampal slices under 60 μg As L^−1^ AsHC 360 exposure. (**A**) Long-term records and representative ED clusters. The boxplots of IIDs and IDs (**B**) amplitude, (**C**) frequency, and (**D**) duration within Pre-, Adding, and Post-stages. “ns” represents no significant difference, “**” indicates *p* < 0.01, and “***” indicates *p* < 0.001.

**Figure 4:**
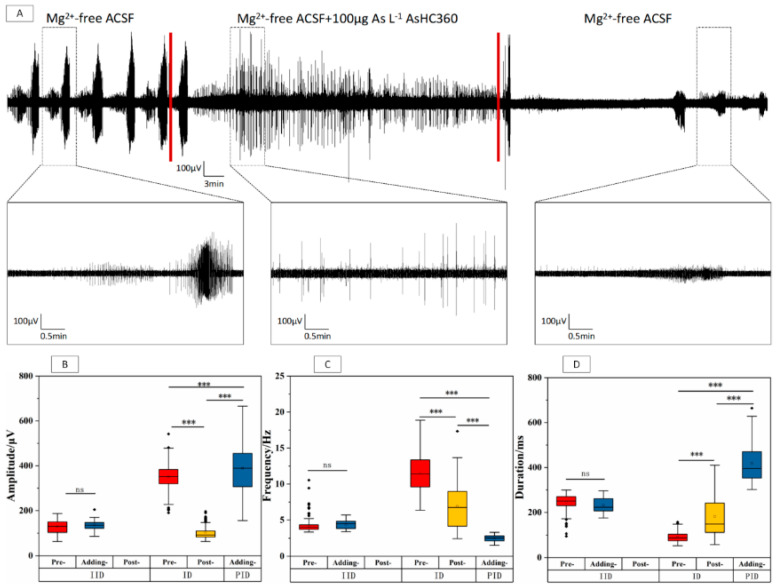
EDs within the CA3 region of hippocampal slices under 100 μg As L^−1^ AsHC 360 exposure. (**A**) Long-term records and representative ED clusters. The boxplots of IIDs, IDs, and PIDs (**B**) amplitude, (**C**) frequency, and (**D**) duration within Pre-, Adding, and Post-stages. “ns” represents no significant difference, “***” indicates *p* < 0.001.

We divided the experimental groups into low- and high-concentration groups, with 5, 20, and 60 μg As L^−1^ comprising the former and 100 and 200 μg As L^−1^ comprising the latter. Figure 2, Figure 3 and Figure 4 shows the EDs signal and corresponding statistical analysis of the experimental groups 2–4, which were exposed to multi-concentration AsHC360 at 20, 60, and 100 μg As L^−1^. The other experimental groups, exposed to AsHC 360 concentrations at 5 and 200 μg As L^−1^, are depicted in Figures S1 and S2, respectively.

The normal EDs clusters can be triggered in the Adding-stage when the hippocampus slices are exposed to AsHC 360 concentrations below 60 μg As L^−1^ (Figures S1–S3), but with different duration, amplitude, and frequency (Table S1). However, when the exposure concentration reached 100 μg As L^−1^, normal EDs could not be formed (Figure 4 and Figure S2). In the Post-stage, the EDs clusters were more affected by AsHC 360. Normal EDs clusters could only be triggered under exposure to 5 μg As L^−1^ AsHC 360 (Figure S1). As the exposure concentration increased, either the EDs formations became highly irregular (20, 60, and 100 μg As L^−1^ AsHC 360, Figure 2, Figure 3 and Figure 4), or no valid EDs could be formed (200 μg As L^−1^ AsHC 360, Figure S2). An interesting point to note is that the clusters of EDs under 100 μg As L^−1^ AsHC 360 exposure disappeared in the Adding-stage but recovered after the elimination (Figure 4).

The results from Figure 2 indicate that exposure to 20 μg As L^−1^ AsHC 360 led to a notable decrease in the amplitude and frequency of IIDs phase. Simultaneously, there was a significant extension in their duration. However, after washing out of AsHC 360, there was no significant difference (* *p* > 0.05) between the Pre- and Post-stages. A similar pattern was observed in the IDs phase, where introducing AsHC 360 led to a significant decrease (* *p* < 0.001) in the amplitude and frequency and a significant increase (* *p* < 0.005) in the duration. On the other hand, eliminating AsHC 360 resulted in a significant increase (* *p* < 0.001) in the amplitude, an elevated inhibition (* *p* < 0.001) of the frequency, and an improvement (* *p* < 0.001) in the duration. These findings suggest that the changes observed in the IIDs phase were short-term and reversible, whereas the changes in the IDs phase had a continuous effect on frequency and duration of EDs.

From Figure 3, the decrement of the amplitude (* *p* < 0.001) and duration (* *p* < 0.01) in the IIDs phase, together with the increment of the frequency (* *p* < 0.01), were found after introducing 60 μg As L^−1^ AsHC 360. These effects disappeared after washing out AsHC 360, since no significant difference (* *p* > 0.05, *n* = 5) existed between the Pre- and Post-stages. Complete reverse effects were observed in the IDs phase compared to the IIDs after adding AsHC 360. Thus, the increment of amplitude (* *p* < 0.001) and duration (* *p* < 0.001) in the IDs phase, together with the decrement of the frequency (* *p* < 0.001) were observed. These effects were reduced but still existed after washing out of AsHC 360 since significant differences between the Pre- and Post-stages were observed. Interestingly, 60 μg As L^−1^ AsHC 360 exposure had a similar short-term and reversible effect on IIDs compared to 20 μg As L^−1^ AsHC 360. In contrast, the impact on the IDs phase is irreversible except for duration.

Based on the results shown in Figure 4, it can be observed that the occurrence of IDs was inhibited, and PIDs were persistently present in the Adding-stage. The characteristics of PIDs discharge were discussed since the nervous system maintains a low excitability level through persistent PIDs/IIDs when it cannot achieve IDs [33,34]. In the IIDs phase, there was no significant change in amplitude, frequency, and duration (* *p* > 0.05, *n* = 4) were found after introducing 100 μg As L^−1^AsHC 360. However, the IIDs phase disappeared after washing out of AsHC 360. In the IDs phase, the amplitude (* *p* < 0.001) and frequency (* *p* < 0.001) significantly decreased, while the duration significantly increased (* *p* < 0.001) in the Post-stage compared to the Pre-stage. A similar trend was observed in the PIDs frequency (* *p* < 0.001) and duration (* *p* < 0.001). The results suggest that the conversion from IIDs/PIDs to IDs was inhibited when exposed to 100 μg As L^−1^ AsHC 360, and the incidence of EDs was significantly reduced. Therefore, complete EDs clusters could not be formed in the Adding-stage. However, this ability was regained in the Post-stage, indicating a reversible effect of AsHC 360 exposure.

The formation of EDs could not be triggered in the Adding-stage, and the ability to form EDs was not regained in the Post-stage when the concentration of AsHC 360 exposure reached 200 μg As L^−1^ (Figure S2).

### 2.3. Agonist Modulates the Effects of AsHC 360 on EDs

**Figure 5:**
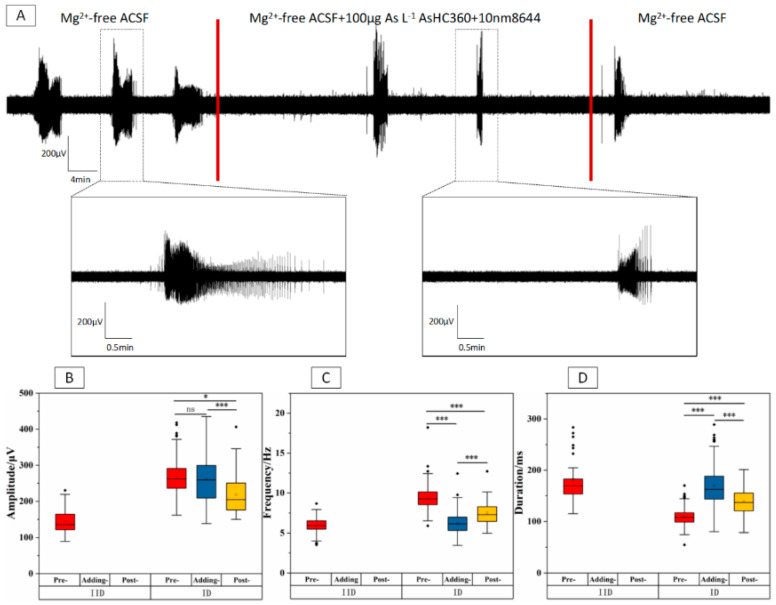
EDs within the CA3 region of hippocampal slices under 10 nM (S)-(-)-Bay-K-8644 and 100 μg As L^−1^ AsHC 360 co-exposure. (**A**) Long-term records and representative ED clusters. The boxplots of IIDs and IDs (**B**) amplitude, (**C**) frequency, and (**D**) duration within Pre-, Adding, and Post-stages. “ns” represents no significant difference, “*” indicates *p* < 0.05, and “***” indicates *p* < 0.001.

We recorded the EDs signals and corresponding statistical analyses of experimental groups 6–7 co-exposed under an L-type calcium channel agonist (S)-(-)-Bay-K-8644agonist (10 nM) and AsHC360 (100 μg As L^−1^) (Figure 5 and Figure S3).

From Figure S3, continuous and enhanced EDs were observed when the hippocampus slices were exposed only to agonists, whereas the formation of a normal EDs signal was observed when co-exposed to both agonist and AsHC 360 (Figure 5).

In Figure 5, there was no significant difference (* *p* > 0.05) in the IDs amplitude in the Adding-stage compared to the Pre-stage, but the ID amplitude was significantly decreased (* *p* < 0.05) in the Post-stage. This indicated that co-exposure of the agonist and AsHC 360 in the Adding-stage could maintain the IDs amplitude, but this effect was not sustainable in the Post-stage. The IDs frequency was significantly reduced (* *p* < 0.001), while the IDs duration was significantly increased (* *p* < 0.001) under co-exposure. These effects slightly recovered in the Post-stage. Although there was a decline in the incidence of EDs, complete and continuous EDs were still maintained.

According to Figure 4, exposure to 100 μg As L^−1^ AsHC 360 can inhibit the formation of EDs signals. Conversely, Figure S3 shows that exposure to 10 nM (S)-(-)-Bay-K-8644 can promote the transfer of IID/PID to IDs. This leads to enhanced EDs signals in the IDs phase but not in the IIDs phase. In Figure 5, the co-exposure to the agonist and AsHC 360 results in normal Eds are shown.

### 2.4. Comparison of Concentration-Dependent Effects on EDs

**Figure 6:**
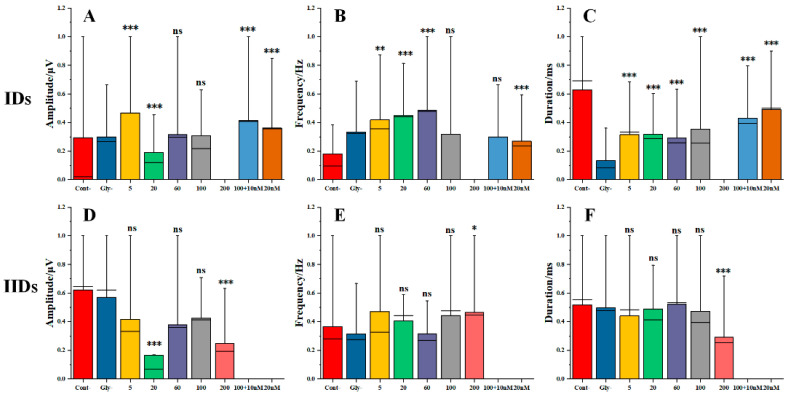
The relative changes of IIDs and IDs parameters due to multi-concentration AsHC 360 exposure and co-exposure using agonist and AsHC 360. (**A**) The relative amplitude of IDs. (**B**) The relative frequency of IDs. (**C**) The relative duration of IDs. (**D**) The relative amplitude of IIDs. (**E**) The relative frequency of IIDs. (**F**) The relative duration of IIDs. “ns” represents no significant difference, “*” indicates *p* < 0.05, “**” indicates *p* < 0.01, and “***” indicates *p* < 0.001.

In this study, we investigated the parameters of IDs and IIDs under various concentrations of AsHC 360 exposure, specifically in the Control group (cout-), the pre-control group containing Glycerol (Gly-), 5 μg As L^−1^ group, 20 μg As L^−1^ group, 60 μg As L^−1^ group, 100 μg As L^−1^ group, and 200 μg As L^−1^ group. Additionally, we examined the effects on IDs and IIDs parameters when co-exposed to excitatory agents at 10 nM and 20 nM (S)-(-)-Bay-K-8644, in the presence of AsHC 360 at a concentration of 100 μg As L^−1^. The data from each group was then normalized to the glycerol data and presented in Figure 6 for comparison. The results are shown in Figure 6.

As illustrated in Figure 6, during the addition phase, AsHC 360 exhibits inconsistent effects on the amplitude and duration of the IDs phase. Its relative frequency demonstrates a trend of initial increase followed by a decrease as the concentration rises. Under identical conditions, an excessively high concentration of AsHC 360 (200 μg As L^−1^) fails to establish a regular IDs phase. The formation of the IIDs phase during the addition stage is largely unaffected by AsHC 360, with a notable change only evident when exposed to a concentration of 200 μg As L^−1^. When brain slices are exposed to excitatory agents, the ID phase of the addition stage can be formed under both conditions (with/without AsHC 360), while the IIDs phase cannot be established.

In the addition phase, the relative amplitude of ID parameters (Figure 6A) exhibits an irregular variation, specifically under the exposure of AsHC 360 at concentrations of 5 (* *p* < 0.001) and 20 μg As L^−1^ (* *p* > 0.001). The relative frequency (Figure 6B) strengthens with increasing concentrations of AsHC 360 from 5 to 60 μg As L^−1^. However, at a concentration of 100 μg As L^−1^ of AsHC 360, the frequency shows no significant difference compared to the glycerol-containing control group (* *p* > 0.05). The duration of the EDs signal under AsHC 360 exposure (Figure 6C) consistently exceeds that of the glycerol-containing control group. The introduction of an excitatory agent diminishes the frequency enhancement caused by AsHC 360 exposure while maintaining the regular occurrence of EDs.

During the IID phase of the addition stage, except at 200 μg As L^−1^, the relative frequency and duration under AsHC 360 exposure (Figure 6E,F) show no significant difference compared to the glycerol-pre-treated control group (* *p* > 0.05). However, when exposed to AsHC 360 at a concentration of 200 μg As L^−1^, both the relative amplitude and duration are significantly lower than the glycerol-pre-treated control group (* *p* < 0.001), while the relative duration is significantly higher than the glycerol-pre-treated control group (* *p* < 0.05).

**Figure 7:**
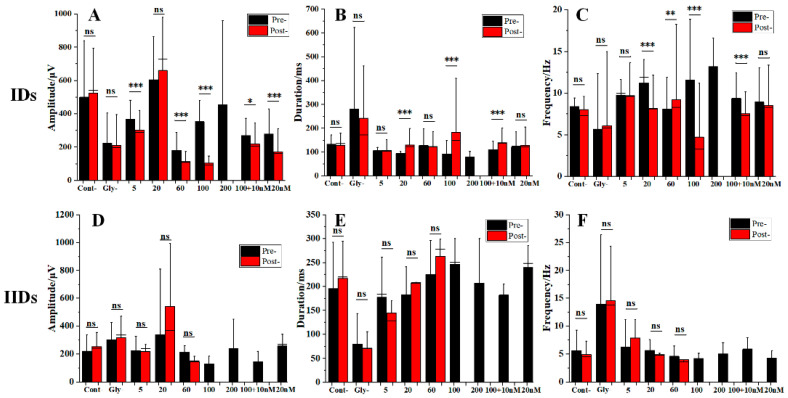
The changes of IIDs and IDs parameters under multi-concentration AsHC 360 exposure and co-exposure with agonist and AsHC 360 between Pre- and Post-stages. (**A**–**C**) are the amplitude, frequency, and duration of IIDs, respectively. (**D**–**F**) represent the amplitude, frequency, and duration of IDs, respectively. “ns” represents no significant difference, “*” indicates *p* < 0.05, “**” indicates *p* < 0.01, and “***” indicates *p* < 0.001.

The comparison of the IIDs and IDs parameters between the Pre- and Post-stages is presented in Figure 7.

From Figure 7, it can be observed that the IDs phase in the Post-stage was more affected by higher AsHC 360 (200 μg As L^−1^), which prevented the formation of a normal IDs phase. However, effective IDs phases were observed at lower AsHC 360 concentrations (below 100 μg As L^−1^). Similarly, the formation of the IIDs phase in the Post-stage was more affected by higher AsHC concentrations (100 and 200 μg As L^−1^), preventing the formation of a normal IIDs phase. Effective IIDs phases were only observed at lower AsHC 360 concentrations (below 60 μg As L^−1^). When brain slices were exposed to an agonist, the IDs phase in the Post-stage was formed under both conditions (with/without AsHC 360), while the IIDs phase could not be created.

Significant changes in the IDs parameters were observed between the Pre- and Post-stage under different AsHC 360 exposure conditions. Specifically, significant decreases in amplitude (Figure 7A) were observed in all conditions except for the 20 μg As L^−1^ exposure. Additionally, there were significant reductions in frequency (Figure 7B) and increases in duration (Figure 7C) under 20 and 100 μg As L^−1^ AsHC 360 exposure. In contrast, for the IIDs parameters, no significant differences were observed between the Pre- and Post-stage under AsHC 360 exposure of 5–60 μg As L^−1^ for amplitude, frequency, and duration (Figure 7E,F). When exposed to an agonist, the IDs amplitudes were lower in the Post-stage than in the Pre-stage under both conditions (with/without AsHC 360). The IDs frequency and duration were also lower in the Post-stage with AsHC 360. Additional experimental results can be found in Supplementary Figures S1–S4, as well as in Tables S1 and S2.

## 3. Discussion

Previous studies suggest that an imbalance in the excitatory and inhibitory neurotransmitters may contribute to the development of seizures. Disruption to this balance, such as increased level of glutamic acid and aspartic acid or decreased level of GABA and Glycine may potentially play a role. However, it is important to note that glutamic acid in the blood cannot penetrate the blood–brain barrier [35]. Instead, glucose entering the cell is converted into glutamic acid through the amino acid transporter system [36]. Glucose transporters (GLUTs) are primarily distributed on the cell membranes of astrocytes or neurons [35,37]. Studies have also investigated the role of specific neurotransmitter receptors in seizure activity. For example, blocking the GABA_A_ receptor has been shown to shorten IIDs, while blocking the glutamate receptor can block IDs [38]. These findings suggest that the balance between inhibitory and excitatory neurotransmitters is crucial for the proper functioning of the brain and the prevention of seizures. The lack of significant effect of AsHC 360 exposure on the IIDs phase of hippocampal EDs suggests that the status of GABA_A_ receptors in hippocampal slices was not affected by the exposure, as reported in a previous study [38].

Previous studies suggest that there was a transition process before the onset of a seizure, and the IID phase could be recognized as a typical epilepsy discharge [39]. Prior to the seizure onset, a decrease in the metabolic rate and neuronal activity in the brain has been found to occur before the onset of temporal lobe seizures [40]. This transition period from IID/PID to ID has been recognized as a reliable reference for epileptic seizure prediction. In our current work, it shows the possibility that the transition period can be modulated by AsHC 360 exposure. In addition, the gradual suppression of the conversion of EDs from IIDs/PIDs to IDs with increasing AsHC 360 concentration was consistent with the inverse relationship between IIDs/PIDs and IDs of EDs reported by Zhang et al. [38].

A plausible explanation for the influence of AsHC 360 exposure on epileptiform discharges could be linked to glutamate receptors. For example, (S)-(-)-Bay-K-8644, a dihydropyrimidine derivative, enhances the opening probability of a specific subtype of L-type Ca^2+^ channel. This action promotes voltage-dependent calcium influx and corrects the expression of glutamate receptor [41,42]. Although no direct study has investigated how AsHCs interact with EDs, analogous research suggests that (1) low concentrations of sodium arsenite (0–1 μM) can increase AMPAR expression, whereas higher concentrations (>1 μM) inhibit the expression of specific subunit glutamate receptor 1 [30]; (2) substantial amount of AsHC 360 (45–150 μg As L^−1^) can disturb nerve cell membrane integrity, subsequently affecting NMDAR and AMPAR activity [15]. Research has shown that exposure to high concentrations of arsenolipids can lead to neurotoxicity [43]. Therefore, in our subsequent studies, we will focus on using tools such as patch-clamps to specifically examine the proportion of cell viability after exposure to high concentrations of Arsenic 360. This will provide a more scientific basis for the rational use of Arsenic 360.

**Figure 8:**
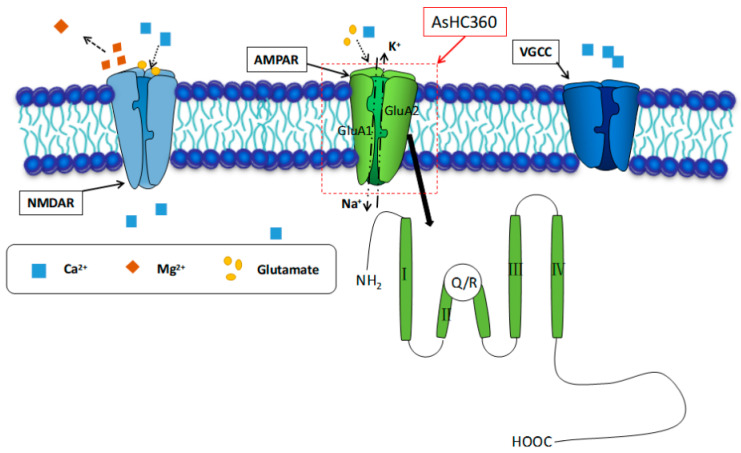
The present situation of the postsynaptic membrane and the possible role of AsHC 360 during magnesium-free induced seizures.

An intriguing finding in our study was the restoration of ED cluster formation when AsHC 360 exposure was combined with an agonist. This combination resulted in controlled AMPAR activity that influenced the transition from IIDs/PIDs to IDs. This suggests that AMPARs play a pivotal role in mediating AsHC 360’s impact on neuronal network dynamics. Consequently, the excessive opening of L-voltage-gated calcium channels in the co-exposure experiment might expedite the transformation from IIDs/PIDs to IDs, thereby mitigating the inhibitory effects of AsHC 360. Previous studies have suggested that AsHCs generally have an inhibitory effect on cells. However, partial concentration AsHCs may promote the expression of glutamate receptors and enhance the intracellular Ca^2+^ concentration in the hippocampus [15,30]. In the current study, exposure to AsHC 360 increased the frequency of EDs at low concentrations but inhibited them at high concentrations. For instance, 20 μg As L^−1^ of AsHC 360 enhances the transformation from IIDs/PIDs to IDs by elevating the expression of the glutamate receptor, thereby promoting EDs. AMPARs, a major subtype of glutamate ionotropic receptors, facilitate Na+ influx and K^+^ efflux when activated. They have a slow onset but extended action durations. The activation of NMDAR channels permits the influx of extracellular Ca^2+^ during seizures triggered by magnesium-free conditions. This action prompts synaptotagmin binding, mediating the fusion of vesicles with the presynaptic membrane and subsequently releasing neurotransmitters. The consistent release and buildup of the excitatory neurotransmitter, glutamic acid, from the presynaptic membrane leads to its binding with ionotropic receptors AMPAR and NMDAR on the postsynaptic membrane. Consequently, the ligand-gated ionotropic channel opens, initiating Ca^2+^ influx into the postsynaptic pyramidal neurons and setting off a cascade of action potentials. Moreover, neuronal glutamic acid release is Ca^2+^-dependent, as shown in Figure 8. Furthermore, to provide a more comprehensive understanding of the underlying mechanisms, we are currently conducting experiments specifically targeting ion channels. These studies are crucial for shedding light on the intricate interplay between AsHC 360 and neuronal activity, particularly in the context of how various ion channels modulate this relationship. Preliminary findings suggest intriguing patterns, but a thorough analysis is underway. By delving deeper into these ion channel dynamics, we aim to further validate and solidify the conclusions drawn in the present study. It is our hope that these additional experiments will offer more insights into the potential therapeutic implications of our findings.

The effects of AsHC 360 exposure on the occurrence frequency of EDs and the amplitude of the IDs phase suggest that the increase in glutamate receptor protein enhanced the interaction of glutamic acid with AMPARs, mainly by promoting the expression of glutamate receptor at low concentrations (<60 μg As L^−1^) of AsHC 360 exposure, thereby accelerating the completion of the action potential. Therefore, the occurrence frequency of EDs increases when exposed to AsHC 360. The amplitude is inversely proportional to the frequency when the total concentration of intracellular and extracellular calcium ions remains unchanged. Regarding lower concentrations of AsHC 360, it would indeed be reasonable to extend the investigation to explore the effects of concentrations below 60 μg As L^−1^. This approach would help establish a comprehensive understanding of the dose–response relationship and provide insights into whether there is a threshold concentration below which AsHC 360 ceases to exert significant effects on the studied parameters. Furthermore, determining the specific sites and mechanisms of AsHC 360 interaction with AMPARs, NMDARs, or other molecular components of the system is a crucial direction for future research. Techniques such as molecular modeling, receptor binding assays, and electrophysiological studies could be employed to shed light on these intricate interactions, unraveling the molecular basis of AsHC 360’s impact on neuronal networks.

Presently, the accumulation of arsenic in various parts of the brain, such as the pituitary gland, hippocampus, striatum, etc., [19] and its ability to pass through the placental barrier and the blood–brain barriers [44] highlights the importance of further studying its effects on the central nervous system, particularly through animal models. While moderate arsenic exposure to AsHCs has been linked to cognitive defects in children, [2,45] the cytotoxicity of AsHC 360 is higher than that of iAs^III^, [46] indicating the need for timely risk assessment of AsHCs exposure, particularly in infancy when brain neurotransmitters are still developing. The findings from this study suggest that the alteration of AMPAR activity due to AsHC 360 exposure could contribute to changes in neuronal excitability, which, in turn, might be linked to the development of neurological disorders. Therefore, future investigations should focus on uncovering the precise mechanisms by which AsHC 360 disrupts AMPAR function and exploring how these disruptions translate to neurobiological and behavioral outcomes.

While our current study predominantly focused on the effects of AsHC on the epileptiform activity using an in vitro model, it is plausible that adult animals might exhibit differential responses due to mature neuronal networks and other age-related factors. However, this is speculative, and direct investigations on adult animals would be required to draw definitive conclusions. Given that epileptic animals already have a heightened neuronal excitability, the response to AsHC might differ from non-epileptic counterparts. Factors like the severity of the epilepsy, the specific origin of the epileptiform activity, and the age of the animal might all influence the effects of AsHC. In addition, it is conceivable that the response to AsHC might vary depending on the specific characteristics and mechanisms underpinning each model. The 4AP model, for instance, predominantly enhances synaptic transmission by blocking potassium channels, which could modulate the effects of AsHC differently than in our current model.

In the realm of contemporary neuropharmacological research, understanding the precise mechanistic role of any agent is pivotal. In our study, while we delved into the effects of AsHC 360, our current data does not conclusively label it as an anti-epileptic agent. Instead, a deeper dive into our findings hints at a nuanced picture where AsHC 360 potentially exhibits an inhibitory effect on synaptic transmission and/or neuronal activity. This observation warrants a broader perspective on its role in the complex neuronal networks and pathways. One constructive approach to unpack this would be to test the compound across different concentrations on brain slices incubated in regular (i.e., Mg^2+^-containing) aCSF. Such an approach is not just about ensuring empirical rigor but is essential to trace the exact modus operandi of AsHC 360 in the neuronal milieu. Given the promising leads and the feedback received, we are inclined towards adopting this strategy in our forthcoming studies, with the anticipation that it would provide a clearer and more comprehensive understanding of AsHC 360’s action.

Overall, establishing an exposure model of AsHCs in vivo assisted by biochemistry and molecular biology, can provide deeper insights into the effects and mechanisms of AsHC on epileptiform activity, memory, and cognitive skills.

## 4. Materials and Methods

### 4.1. Chemicals, Reagents, and Standards

Purified water (≥15 MΩcm) was procured from Elix Essential 5 system (Millipore SAS, 67,120 Molsheim, France). All the reagents were of analytical grade with a purity greater than 99% (Tianjin Kemeo Chemical Reagent Co., Ltd., Tianjin, China). Acutely isolated rat hippocampal brain slices were incubated with ACSF having concentrations of 10 mM Glucose, 1.3 mM MgCl_2_, 124 mM NaCl, 2 mM CaCl_2_, 1.2 mM NaH_2_PO_4_·2H_2_O, 25 mM NaHCO_3_, and 3.5 mM KCl [47]. The Mg^2+^-free ACSF (pH7.4) without MgCl_2_-induced EDs in brain slices. L-type calcium channel agonist (S)-(-)-Bay-K-8644 was obtained from MCE (Med Chem Express LLC, Shanghai, China). Solid AsHC 360 containing 2 μM As was provided by the central laboratory in Graz, Austria. It was dissolved in Glycerin/H_2_O (4/1, *v*/*v*) and diluted to different concentrations of 5, 20, 60, 100, and 200 μg As L^−1^. The diluted AsHC 360 solution was packaged in 2 mL centrifuge tubes, stored at 0 °C, and dissolved in 50 mL of Mg^2+^-free ACSF with 95% O_2_/5% CO_2_ mixed with a blender before use [48].

### 4.2. Animals and Hippocampal Slices Preparation

A total of 15 Sprague Dawley male rats (8–12 days) were provided by the Beijing Weitong Lihua Experimental Animal Technology Co., Ltd. (Certification No.: SCXK 2021-0006). The rats were housed in the animal room under standard experimental conditions (12 h light/dark cycle, 23–25 °C, water, and food ad libitum). Collection of animal tissue samples for this study was approved as part of the study protocol. This animal study was approved by Institutional Animal Care and Use Committee of Tiangong University (Tianjin, China)—approval: IRM-DWLL-2022124. All animal housing and experiments were conducted in strict accordance with the institutional Guidelines for Care and Use of Laboratory Animals.

The rats were retrieved and weighed one hour before the experiment. Chloral hydrate was injected intraperitoneally with a concentration of 10%, depending on the 0.4 mL/100 g standard [49,50,51]. After deep anesthesia, the rats’ brains were removed, clamped using ice clips, fixed with liquid agar in the mold, and sliced horizontally (400 μm thickness) in ice-cold artificial cerebrospinal fluid with 95% O_2_/5% CO_2_ mixed gas by a vibrating tissue slicer (VF-2000, Precisionary Instruments, Inc., Greenville, NC, USA). The horizontal brain slices encompassing the ventral hippocampal areas with intact hippocampal CA1, CA3, and DG regions were selected and transferred into an incubation tank with artificial cerebrospinal fluid at 32 °C for at least 2 h.

### 4.3. Epileptiform Activity Induction

The multi-electrode array electrophysiological recording system comprises an MEA host (MEA2100-60, Reutlingen, Germany), perfusion system (PPS2 1.3.2, Multi-Channel Systems, Germany), and temperature controller (TC02, Multi-Channel System, Germany) [15]. The incubated hippocampal brain slice was quickly transferred from the incubation tank during the experiment to the glass electrode of MEA2100-60. Then, the glass electrode was placed in MEA2100-60, the position of the hippocampal brain slice was adjusted, and the corresponding positions for each division of the hippocampal brain slice and the 64 recording points of the glass electrode were determined. The mixed gas containing 95% O_2_/5% CO_2_ was continuously introduced into ACSF 10 min before the perfusion system started. This was done to ensure the activity of brain slices and the normal metabolic capacity. Simultaneously, the temperature controller was turned on to maintain the MEA2100 recording platform and the perfusate at 37 °C. In the experiment, continuous perfusion of Mg^2+^-free ACSF elevated the opening degree of the NMDAR channel by weakening the blocking effect of Mg^2+^ on NMDAR channel, [52] inducing the synchronous firing activity of hippocampal neurons. Abnormal synchronized EDs in pyramidal cell layers of CA1, CA3, and DG hippocampus regions were recorded after 10 to 15 min and gradually entered a steady state. In this study, epileptiform events with a continuous discharge time of less than 300 ms were characterized as IIDs (inter-ictal discharges). Moreover, epileptiform events having a constant discharge time of 300–2000 ms were defined as PIDs (pre-ictal discharges). Additionally, epileptiform events with continuous discharge times more than 2000 ms were identified as IDs (ictal discharges) [53]. In this study, we also adopted a minimum frequency of activity criterion to exclude events characterized by low-frequency spiking that are not indicative of genuine epileptiform activity.

### 4.4. Experimental Protocol

**Figure 9:**
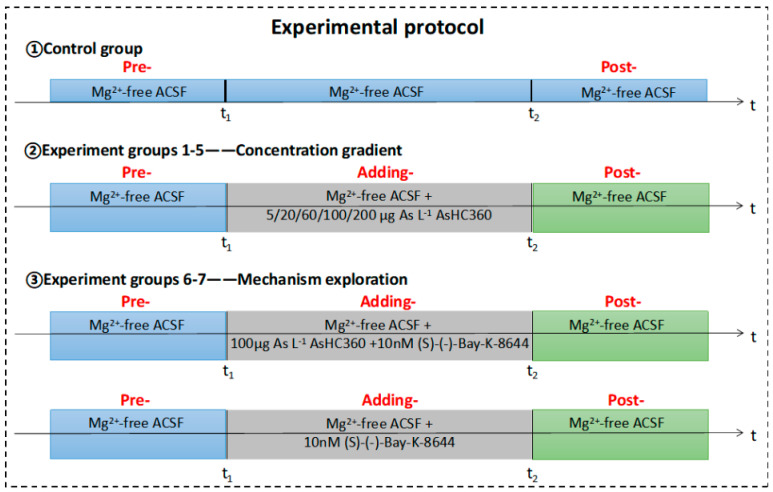
The experimental protocol. t_1_: introducing AsHC 360 (with/without agonist) in ACSF, t_2_: washing out AsHC 360 and/or agonist by adding Mg^2+^-free ACSF.

The experimental protocol is depicted in Figure 9. The control group recorded the whole ED process induced by Mg^2+^-free ACSF. The experimental groups 1–5 were exposed to multi-concentration AsHC 360 at 5, 20, 60, 100, and 200 μg As L^−1^ concentrations. The experimental groups 6–7 were exposed to an agonist of 10 nM (S)-(-)-Bay-K-8644 together with/without AsHC 360 (100 μg As L^−1^). In Pre-stage EDs, signals were recorded for at least 20 min to obtain a stable status. However, an addition was introduced in the Adding-stage in Mg^2+^-free ACSF at t_1_, and the EDs signals were continuously recorded until t_2_ (time to wash out addition). This stage lasts longer than 40 min (from the starting point of additive addition to the starting time of wash-out). In the Post-stage, the ACSF was replaced using AsHC 360 free ACSF to eliminate AsHC 360 and/or agonist, lasting 30 min. EDs will differ due to the individual differences between experimental samples and the subtle difference when recording EDs. In our study, we conducted multiple experiments (*n* > 3) under the same conditions within each group and recorded EDs. To ensure consistency and account for individual variations, we selected one representative experimental result from each group for inclusion in the article. In the data analysis section, we calculated the average values of each parameter based on all recorded EDs under each experimental condition. For statistical processing, at least three ED signals were recorded from each stage.

### 4.5. Statistics

The study used the MC_Rack software (MC_Rack 4.6.2, Multi-Channel Systems, Kusterdingen, Germany) for data acquisition and processing, setting the bandwidth of the band-pass filter to 10–5000 Hz. The adoption rate was 20 kHz, using four times the standard deviation of noise as the threshold, and we recorded the local field potential of EDs by each electrode. Origin 9.0 (origin lab, Northampton, MA, USA) and MATLAB (math Works Inc., Natick, MA, USA) were utilized for data recording and statistical analysis. Significant differences were analyzed between groups using one-way analysis of variance (ANOVA) and Tukey’s posterior. The statistical results were expressed as Mean ± Standard Deviation (SD). The observed differences were statistically significant at * *p* < 0.05, ** *p* < 0.01, and *** *p* < 0.001; ns is not statistically significant.

## 5. Conclusions

The results suggest that AsHC 360 exposure can affect the formation of ED clusters and ID parameters in a concentration-dependent manner. High levels of AsHC 360 were found to inhibit the formation of ED clusters and affect the IDs phase in the Adding-stage, but not the IIDs phase. Furthermore, the amplitude of the ID was inhibited under AsHC 360 exposure in all concentrations except for 20 μg As L^−1^. However, the relative frequency of ID parameters in the Adding-stage showed concentration-dependent phenomena under AsHC 360 at concentrations below 100 μg As L^−1^.

Co-exposure to an agonist and AsHC 360 was found to restore the ability of EDs cluster formation by controlling AMPARs to regulate the transformation from IIDs/PIDs to IDs. These findings suggest that AsHC exposure may affect the regulation of AMPARs and subsequently alter neuronal excitability, leading to potential neurological disorders. The current study lays the foundation for future research endeavors aiming to unravel the intricate interactions between AsHC 360 and neuronal network components. Further research is needed to elucidate the exact mechanisms underlying the effects of AsHC exposure on AMPARs and EDs formation. Understanding these mechanisms will not only contribute to our knowledge of the neurological impacts of arsenic exposure but also pave the way for potential therapeutic interventions to mitigate its adverse effects on neural circuitry and human health.

## Data Availability

The data supporting the findings of this study are available upon request.

## Supplementary Materials

The supporting information can be downloaded at: https://www.mdpi.com/article/10.3390/ijms242316806/s1.
