# Supplementary material for: AsHC 360 Exposure Influence on Epileptiform Discharges in Hippocampus of Infantile Male Rats In Vitro
Source: Int J Mol Sci. 2023 Nov 27;24(23):16806. doi: 10.3390/ijms242316806 (PMC10705907; doi:10.3390/ijms242316806)
Supplement: Supplementary file 1 [file ijms-24-16806-s001.zip › ijms-2614313-SI.pdf]

Supporting Information

Figure S1. EDs in the CA3 region of hippocampal slices under Glycerin/H_2O exposure

Figure S2. EDs in the CA3 region of hippocampal slices under 5 μg As L^{-1} AsHC 360 exposure

Figure S3. EDs in the CA3 region of hippocampal slices under 200 μg As L-1 AsHC 360 exposure

Figure S4. EDs in the CA3 region of hippocampal slices under 10 nM (S)-(-)-Bay-K-8644 exposure

Table S1. Discharge characteristics of EDs phases (IIDs and IDs)

Table S2. The occurrence frequency/time of epileptic-like events at different stages for various concentrations

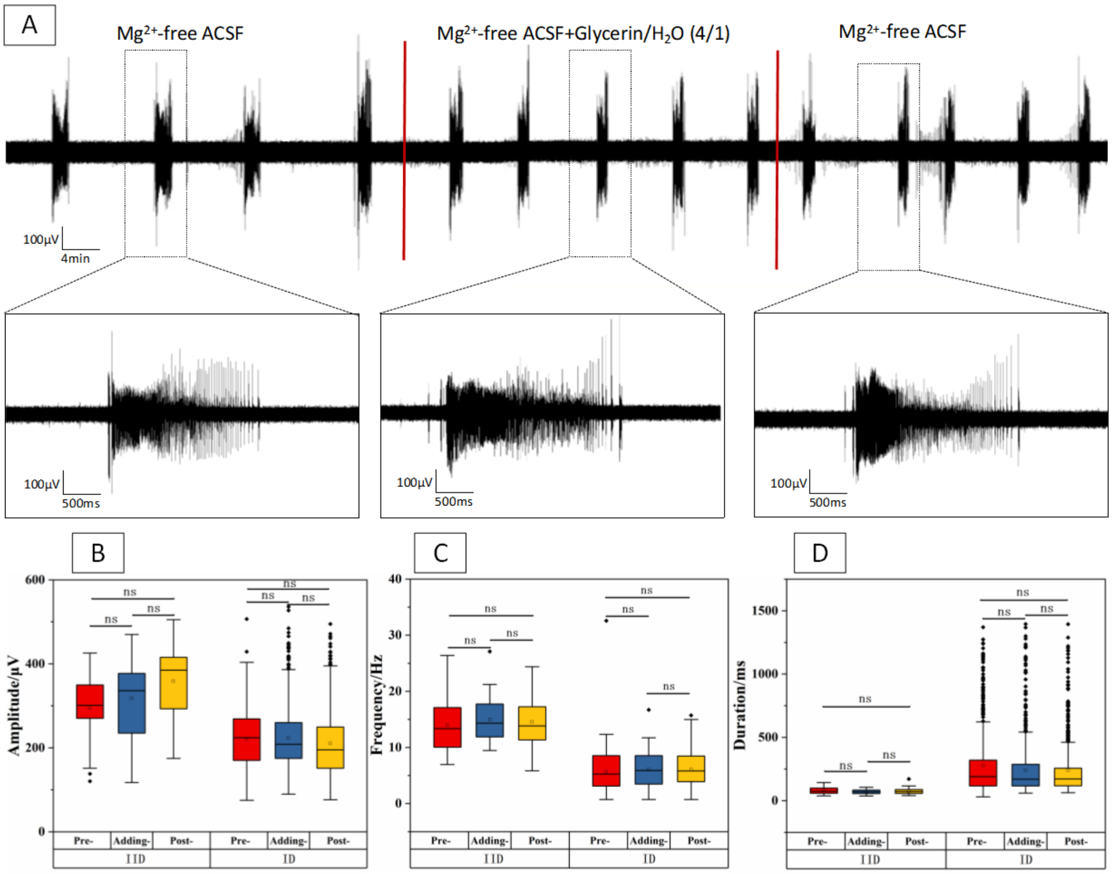

Figure S1. EDs in the CA3 region of hippocampal slices under Glycerin/H_2O exposure. (A) Long-term records and representative ED clusters. Boxplots of IIDs and IDs (B) amplitude, (C) frequency, and (D) duration in Pre-, Adding, and Post- stages.

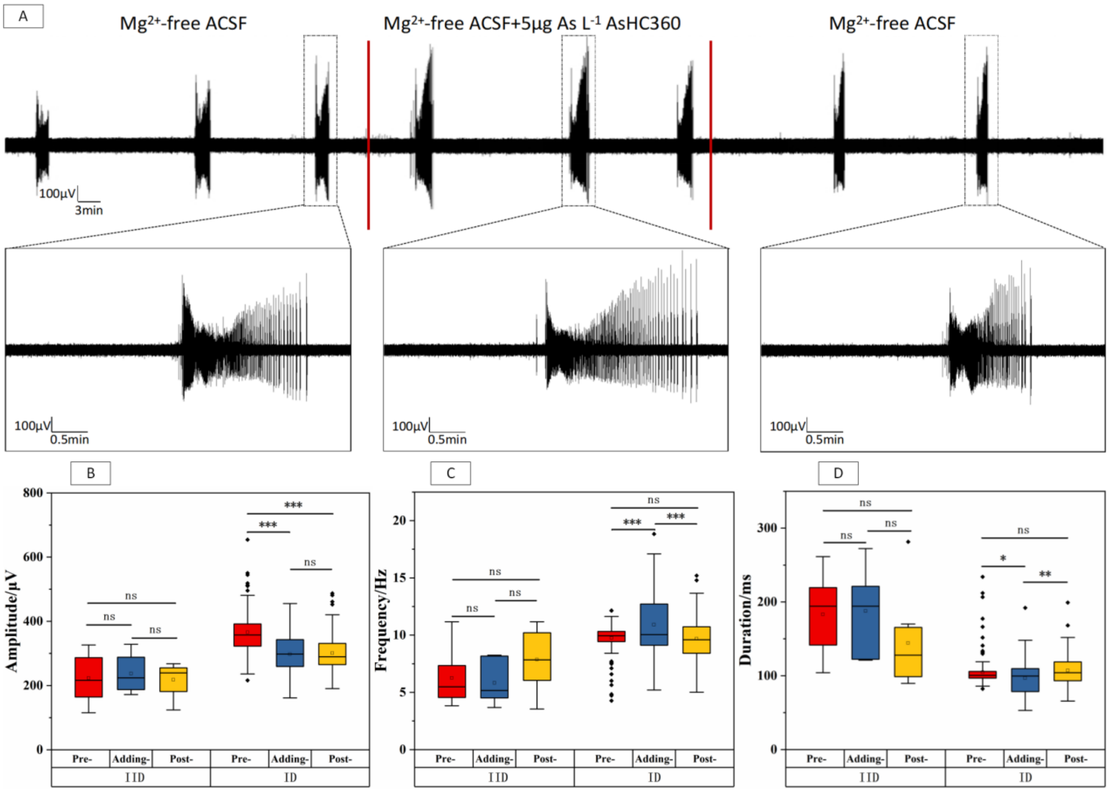

Figure S2. EDs in the CA3 region of hippocampal slices under 5 μg As L^{-1} AsHC 360 exposure. (A) Long-term records and representative ED clusters. Boxplots of IIDs and IDs (B) amplitude, (C) frequency, and (D) duration in Pre-, Adding, and Post- stages.

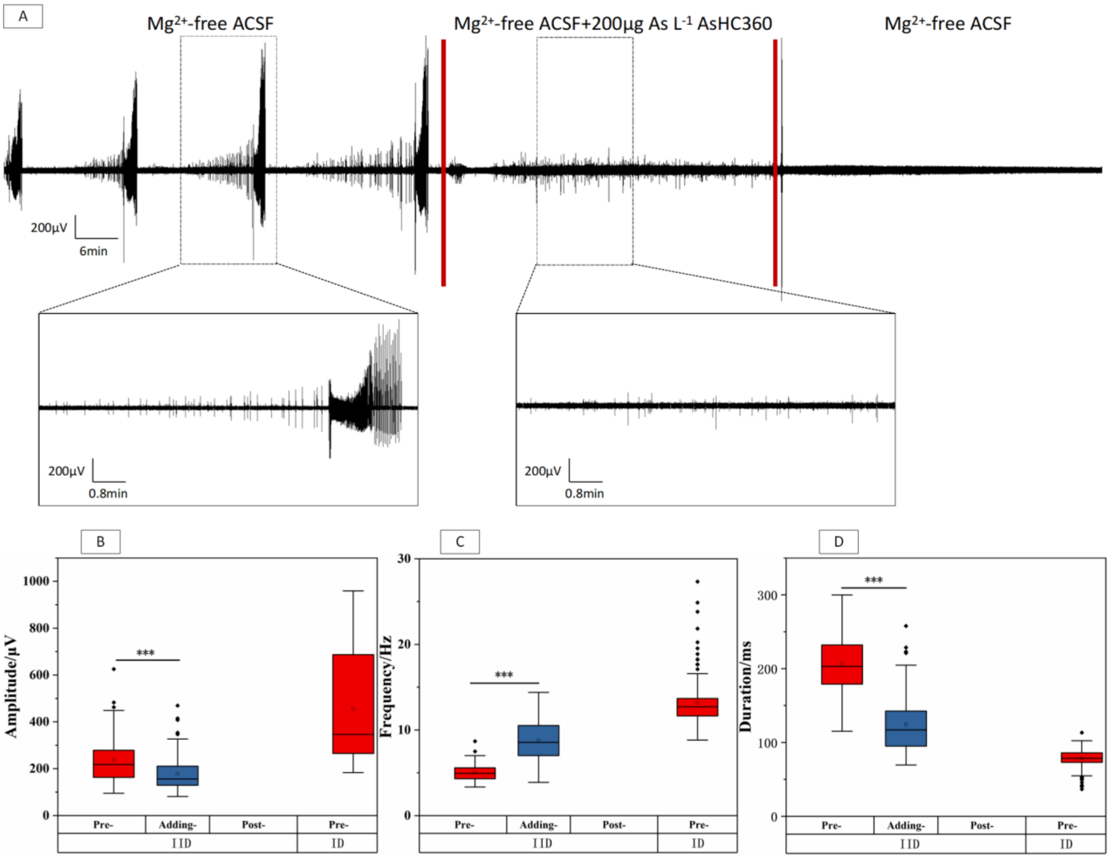

Figure S3. EDs in the CA3 region of hippocampal slices under 200 μg As L^{-1} AsHC 360 exposure. (A) Long-term records and representative ED clusters. Boxplots of IIDs and IDs (B) amplitude, (C) frequency, and (D) duration in Pre-, Adding, and Post- stages.

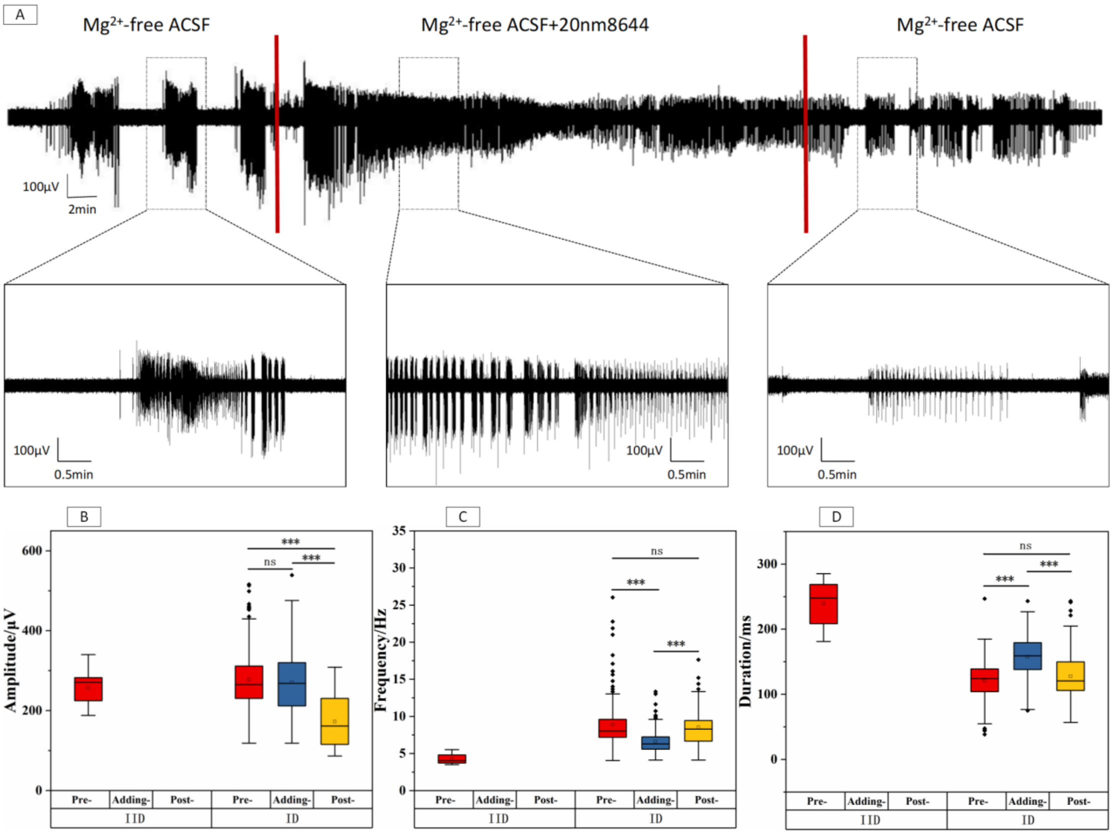

Figure S4. EDs in the CA3 region of hippocampal slices under 10 nM (S)-(-)-Bay-K-8644 exposure. (A) Long-term records and representative ED clusters. Boxplots of IIDs and IDs (B) amplitude, (C) frequency, and (D) duration in Pre-, Adding, and Post- stages.

Table S1. Discharge characteristics of EDs phases (IIDs and IDs). Including the control group and 5 AsHC 360 exposure groups. (The horizontal line indicates that there is no corresponding status and data).

Characteristics		Stages	Control	5 μg As L^{-1}	20 μg As L^{-1}	60 μg As L^{-1}	100 μg As L^{-1}	200 μg As L^{-1}
IIDs	Amplitude (μV)	Pre-	218.89±60.48	223.094±71.86	338.79±162.21	214.53±81.05	128.65±31.41	237.34±91.77
		Adding-	250.19±45.62	237.27±62.48	216.16±109.05	107.26±19.90	136.64±26.81	177.77±72.03
		Post-	251.64±82.23	217.94±54.98	296.93±79.40	147.76±38.42	suppressed	suppressed
	Frequency (Hz)	Pre-	5.59±1.71	6.26±2.21	5.63±1.01	4.60±0.87	4.20±0.94	5.02±0.88
		Adding-	4.64±1.01	5.82±1.94	4.78±0.75	5.90±1.28	4.40±0.69	8.76±2.47
		Post-	4.93±1.32	7.86±2.66	4.78±1.00	3.98±1.11	suppressed	suppressed
	Duration (ms)	Pre-	194.96±56.85	183.10±48.27	182.72±30.10	224.73±41.68	245.89±36.45	206.73±40.34
		Adding-	224.58±45.81	187.63±58.94	214.16±34.10	175.69±32.52	232.61±37.43	124.36±39.17
		Post-	216.93±55.51	144.44±62.91	207.94±42.82	263.24±54.12	suppressed	suppressed
IDs	Amplitude (μV)	Pre-	500.05±136.32	365.66±62.08	602.91±178.44	179.76±38.86	350.39±49.8	455.13±223.49
		Adding-	509.72±133.79	297.78±64.56	474.27±225.60	373.77±115.41	388.79±118.98	suppressed
		Post-	525.41±117.09	301.03±63.99	659.88±233.93	112.93±27.93	104.17±31.04	suppressed
	Frequency (Hz)	Pre-	8.19±2.58	9.75±1.16	11.19±2.01	8.16±1.62	11.55±2.48	13.18±2.65
		Adding-	8.42±3.50	10.91±2.62	10.04±2.11	5.94±1.29	2.48±0.45	suppressed
		Post-	8.70±3.69	9.69±1.87	8.16±1.79	9.17±3.14	6.87±3.09	suppressed
	Duration (ms)	Pre-	129.44±25.02	104.65±18.97	94.17±28.25	127.22±24.95	90.82±20.59	78.19±12.28
		Adding-	131.45±32.67	96.89±23.19	104.52±24.72	177.52±46.02	418.98±87.29	suppressed
		Post-	128.11±33.15	107.16±21.73	128.68±29.75	122.79±45.68	182.14±91.62	suppressed

Table S2. The occurrence frequency/time of epileptic-like events at different stages for various concentrations (where N and n represent the event counts greater than 20 times respectively).

Groups		Total frequency (Hz) count/time.		
		Pre-	Adding-	Post-
EDs	Control	4/23	5/23	5/23
	5 μg As L^{-1}	3/52	3/50	2/38
	20 μg As L^{-1}	3/30	5/38	3/26
	60 μg As L^{-1}	4/26	3/50	4/41
	100 μg As L^{-1}	5/26	1/51	3/42
	200 μg As L^{-1}	3/56	0/44	0/40
	100ugAsL^{-1}+10nM (S)-(-)-Bay-K-8644	3/36	2/32	1/24
	20nM (S)-(-)-Bay-K-8644	3/20	N/37	n/31
